# Elevated Th17 cell proportion, related cytokines and mRNA expression level in patients with hypertension-mediated organ damage: a case control study

**DOI:** 10.1186/s12872-022-02698-3

**Published:** 2022-06-08

**Authors:** Zhuoqun Wang, Jiannan Wang, Pengfei Yang, Xiwen Song, Yongle Li

**Affiliations:** 1 Department of Cardiology, Tianjin Medical University General Hospital, Tianjin Medical University, Tianjin, 300052 China; 2 Tianjin First Central Hospital, Tianjin, China

**Keywords:** Hypertension, Th17 cells, Interleukin-17

## Abstract

**Background:**

Immune abnormalities and inflammatory responses play critical roles in progression of hypertension. Basic studies have confirmed that Th17 cell and related cytokines are important in promoting hypertension-mediated organ damage, but few clinical evidences have been published. Therefore, our study aimed to investigate the relationship between Th17 cell and its related cytokines and hypertension-mediated organ damage in human.

**Methods:**

This study enrolled 179 patients with hypertension (including 92 with hypertension-mediated organ damage and 87 without hypertension-mediated organ damage) and 63 healthy participants. The proportion of Th17 cells in peripheral blood mononuclear cells was measured by flow cytometry. The concentrations of interleukin-17 and interleukin-23 were detected by enzyme-linked immunosorbent assay. Real time-polymerase chain reaction was used to detect the mRNA expression levels of interleukin-17, retinoic acid-related orphan receptor (ROR) γt and signal transducer and activator of transcription-3 (STAT-3).

**Results:**

The proportion of Th17 cells, the concentration of interleukin-17 and interleukin-23 and the mRNA expression levels of interleukin-17, retinoic acid-related orphan receptor γt and signal transducer and activator of transcription-3 were significantly increased in hypertension-mediated organ damage group compared with those in non-hypertension-mediated organ damage group and control group (*P* < 0.005).

**Conclusion:**

Th17 cells and their associated cytokines may be involved in hypertension-mediated organ damage formation and may be able to serve as new biomarkers of hypertension-mediated organ damage and potential therapeutic targets.

## Background

Hypertension is a major public health issue worldwide and an important risk factor for stroke, myocardial infarction, renal failure and heart failure. Asymptomatic hypertension-mediated organ damage (HMOD) of hypertension, which was called "target organ damage" before, is an intermediate stage of cardiovascular disease and an important predictor of cardiovascular risk. 2018 ESC/ESH Guidelines for the management of arterial hypertension states that any HMOD found in patients with hypertension should be classified as a high-risk group [1]. Therefore, studying the mechanism of the development and progression of HMOD of hypertension is of great significance for its prevention and treatment.

In recent years, several basic studies have confirmed the important role of T cells and their cytokines in animal models of hypertension as well as HMOD, of which Th17 cells and their related cytokines interleukin (IL) -17 and IL-23 have received widespread attention [2–6]. A study at the animal level by Madhur et al. showed that IL-17 produced by T cells plays a key role in the process of angiotensin II (AngII) -induced hypertension (3). In addition, IL-17 / IL-23 deficiency can promote kidney damage induced by deoxycorticosterone acetate (DOCA) + Ang II in hypertensive mice, suggesting that IL-17 plays a critical role in HMOD (2). However, few large-scale population studies have been reported. Therefore, we propose the hypothesis that the inflammatory response mediated by Th17 cells and their cytokines may be one of the mechanisms leading to HMOD in human.

This study intends to detect the proportion of Th17 cells in peripheral blood, the concentrations of IL-17 and IL-23 in the plasma, the mRNA expression level of IL-17, and the mRNA expression levels of retinoic acid-related orphan receptor (ROR) γt and signal transducer and activator of transcription-3 (STAT-3), the key transcription factors of Th17 cells, to explore the possible relationship between Th17 and its cytokines and HMOD, with a view to explaining the mechanism of immune abnormalities in HMOD from the perspective of changes in the number and function of Th17 cells. This will provide preliminary population evidence for immune disorder of HMOD, and provide new potential therapeutic targets for the prevention and treatment of HMOD.

## Methods

### Study population and design

From September 2015 to September 2017, 179 patients with essential hypertension hospitalized in the Department of Cardiology, Tianjin Medical University General Hospital were divided into two groups based on the presence or absence of HMOD, of which 92 were in the HMOD group of hypertension and 87 cases without HMOD. The control group consisted of 63 healthy subjects receiving health examination at our hospital during the same period, including medical history collection, physical examination, blood routine test, urine routine test, stool routine test, liver function, renal function, electrolyte, coagulation function, fasting blood glucose, lipids, thyroid function, cancer biomarkers, electrocardiogram, echocardiography, abdominal ultrasound, thyroid ultrasound, chest computed tomography and fundus photography. All of the subjects in control group underwent HMOD screening and found no abnormalities. All included subjects provided their written informed consent under a protocol approved by the Ethics Committee of Tianjin Medical University, Tianjin, China. The demographic and hypertension data of each group was shown in Table 1.

**Table 1:** Demographic and hypertension data of participants

	Control group (n = 63)	non-HMOD group (n = 86)	HMOD group (n = 92)
Age (mean ± SD, years)	57.3 ± 10.2	59.5 ± 9.1	60.1 ± 7.3
Male (%)	36 (57.1)	45 (51.7)	52 (56.5)
SBP(mmHg)	117 ± 7	150 ± 12*	151 ± 10*
DBP(mmHg)	67 ± 6	92 ± 7*	93 ± 8*
Hypertension history (years)	0	9 ± 4*	10 ± 5*

Demographic and hypertension data in 3 groups were compared by one-way analysis of variance. Bonferroni correction was used for multiple comparisons.

*SD* standard deviation, *SBP* systolic blood pressure, *DBP* diastolic blood pressure, *HMOD* hypertension-mediated organ damage

*P < 0.005 when comparing with control group by Student’s t-test

### Definition and assessments of HMOD

HMOD was defined as the detection of left ventricular hypertrophy, vessel damage or kidney damage. All the following methods and cut-off points were recommended by 2013 ESH/ESC Guideline for hypertension [7].

Left ventricular hypertrophy was detected through electrocardiographic (Sokolow–Lyon index > 3.5 mV, RaVL > 1.1 mV or Cornell voltage duration product > 244 mV*ms) and echocardiographic (left ventricular mass index (LVMI) > 115 g/m^2^ for men or > 95 g/m^2^ for women). NIHON KOHDEN ECG-1250P was used for electrocardiographic scan. PILLIPS IE33 ultrasound machines were used to measure interventricular septal thickness (IVST), left ventricular end diastolic diameter (LVDd) and posterior wall thickness (PWT) on parasternal long axis view for 3 cardiac cycles, followed by calculating the mean value. Body surface area (BSA) was calculated using Stevenson formula [8]: BSA (m^2^) = 0.0061 × height (cm) + 0.0128 × weight (kg) − 0.1529. Left ventricular mass (LVM) was calculated using Devereux formula [9]: LVM (g) = 1.04 × [(LVDd + IVST + PWT)^3^ − LVDd^3^] − 13.6. LVMI (g/m^2^) = LVM/BSA.

Vessel damage was detected through aortic intima-media thickness (IMT), aortic plaque or ankle-brachial index (ABI). IMT and aortic plaque were measured by PILLIPS IE33 ultrasound machines. IMT was defined as the distance between aortic intima and media. IMT was measured on 10 mm distal to common carotid arteries of both sides, followed by calculating the mean value. At the same time, common carotid, internal carotid and external carotid arteries were examined to detect plaque. ABI was calculated as the ratio of the higher of the two systolic pressures (from posterior tibial and dorsalis pedis) at the ankle to the higher of the right and left brachial artery pressures. Vessel damage was defined as detection of IMT > 0.9 mm, aortic plaque or ankle-brachial index < 0.9.

Kidney damage was defined as chronic kidney disease (with estimated glomerular filtration rate (eGFR) 30–60 ml/min/1.73 m^2^) or microalbuminuria (with urinary microalbumin 30-300 mg/24 h or albumin-creatinine ratio 30–300 mg/g) [10]. Urinary creatinine was measured using alkaline picric acid method (Roche Diagnostics, Roche/Hitachi Analyzer, Tokyo, Japan). eGFR was calculated using the improved Chinese population MDRD formula [11]. Urinary microalbumin were measured using immunoturbidimetric method (Roche Diagnostics, Roche/Hitachi Analyzer, Tokyo, Japan).

### Exclusion criteria

Patients were excluded from the study if they met any of the following criteria: Cerebrovascular disease (ischemic stroke, cerebral hemorrhage or transient ischemic attack), heart disease (myocardial infarction, angina pectoris, coronary revascularization, heart failure, severe valvular disease and congenital heart disease), secondary hypertension, eGFR < 30 ml/min/1.73 m^2^, peripheral vascular disease, severe retinopathy (bleeding or exudation, papillary edema) and severe non-cardiovascular disease (infection, connective tissue disease, tumor and surgery, etc.). Common causes of secondary hypertension were tested though medical history collection, physical examination, renal ultrasound, renal artery ultrasound, adrenal CT scan, Aldosterone–renin ratio (correction of hypokalemia and withdrawal of drugs affecting RAA system), plasma cortisol, 24-h urinary cortisol excretion, urinary vanillylmandelic acid, rheumatism antibody, anti-nuclear antibody, anti-neutrophil cytoplasmic antibodies and polysomnography.

### Sample collection

Urine samples were taken early in the morning and stored at − 20 °C until testing. Fast blood of all the subjects were collected from median cubital veins within 24 h of admission. After centrifuging at 1500 r/min for 10 min, blood plasma was isolated from the heparinized blood sample and stored at − 80 °C for enzyme-linked immunosorbent assay (ELISA) and real time-polymerase chain reaction (RT-PCR) tests. Peripheral blood mononuclear cells (PBMCs) were isolated from the rest blood by adding Lymphocytes Separation Medium (Biyuntian, Shanghai, China) and centrifuging at 2200 r/min for 20 min. The cells were cultured in RPMI-1640 medium (Baoxin, Tianjin, China) containing fetal bovine serum, 100 U/ml of penicillin, 100ug/ml of streptomycin, 5958 mg/L of HEPES (Baoxin, Tianjin, China), 50 ug/L of phorbolmyristate acetate (PMA), 1umol/L of Ionomycin and 500ug/L of Brefeldin A (BFA) for 6 h at 37 °C under a 5% CO_2_ environment.

### Detection of Th17 cells by flow cytometry

After centrifuging at 1500 r/min for 5 min, cells were isolated and washed in phosphate buffered saline (PBS). After added with fluorescein isothiocyanate (FITC)-conjugated anti-CD4 and allophycocyanin (APC)-conjugated anti-CD3 (BD PharMingen, New Jersey, America), the cells were incubated at 4 °C for 30 min. Intracellular staining was then performed with the BD Cytofix/Cytoperm Plus fixation/permeabilization solution kit following the manufacturer’s instructions (BD Biosciences) and using phycoerythrin (PE)-conjugated anti-IL-17A (BD PharMingen, New Jersey, America). Flow cytometric analysis was performed to detect Th17 cells. Results were presented as total number of IL-17A + cells among the CD3 + CD4 + .

### Measurement of IL-17and IL-23 by ELISA

IL-17 and IL-23 were measured in plasma samples by ELISA using commercial kits (R&D Systems, America) on a microplate reader (Model 680; Bio-Rad) according to the manufacturer's instructions.

### Measurement of mRNA level of IL-17, RORγt and STAT-3 by RT-PCR

Total mRNA was extracted from PBMCs samples using TRIzol reagent according to the manufacturer's recommendations. An RT-PCR kit from TaKaRa was used for complementary DNA (cDNA) synthesis. The levels of mRNA expression of IL-17, RORγt and STAT-3 were quantified using SYBR Green method and a 7500 real-time PCR system. The sequences of the primers are presented in Table 2. The 2^−ΔΔ*C*t^ method was used to measure the relative expression of the target genes compared with the β-actin as the housekeeping control.

**Table 2:** Primer sequences used in RT-PCR experiments

mRNA	Primer sequences (5' → 3')	Amplicon size (bp)
RORγt	Forward CTCCATCTTTGACTTCTCCCACTCCCTA	247 bp
	Reverse CACATGCTGGCTACACAGGCTC	
β-actin	Forward CTGGAACGGTGAAGGTGACA	139 bp
	Reverse AAGGGACTTCCTGTAACAATGCA	
STAT3	Forward ACCTGCAGCAATACCATTGAC	122 bp
	Reverse ACCTGCAGCAATACCATTGAC	
IL-17	Forward GAATTCCGGCAGGCACAAAC	273 bp
	Reverse AAGGTGAGGTGGATCGGTTG	

*bp* base-pair, *RORɣt* retinoic acid-related orphan receptor γt, *STAT 3* signal transducer and activator of transcription-3, *IL* interleukin

### Statistical analysis

Statistical analysis was performed using SPSS 21.0 software (IBM, Somers, NY). Measurement data with normal distribution were expressed as mean ± standard deviation (SD). The t-test was used to compare the means between the two groups. One-way analysis of variance (ANOVA) was used to analyze the means between multiple groups, and Bonferroni correction was used for multiple comparisons. *P* < 0.05 was considered statistically significant.

## Results

### Th17 cell frequency was significantly increased in HMOD patients

**Fig. 1:**
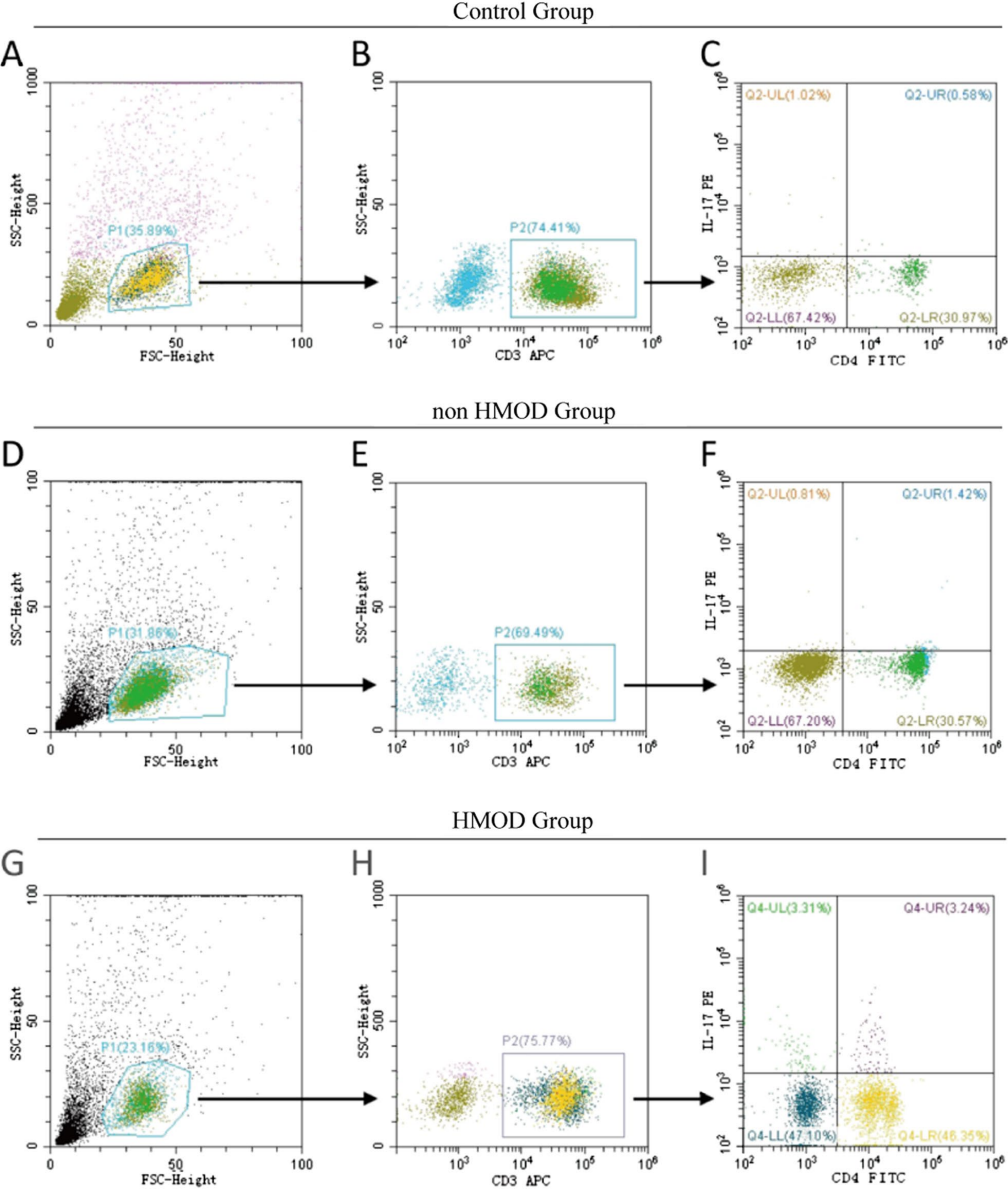
The proportion of Th17 cells revealed by flow cytometry analysis. **A**–**C** The proportion of Th17 cells in control group. P1: lymphocytes. P2: CD3 + lymphocytes. The percentage of CD3 + CD4 + IL-17A + (Th17) cells among the total CD3 + CD4 + T cells was indicated in the upper right quadrant of **C**. **D**–**F** and **G**–**I** shows the proportion of Th17 cells in non-HMOD group and HMOD group, respectively. The proportion of Th17 cells was significantly increased in HMOD and non-HMOD group compared with control group, while Th17 cells was also increased in HMOD group compared with non-HMOD group (*P* < 0.005). HMOD, hypertension-mediated organ damage

**Fig. 2:**
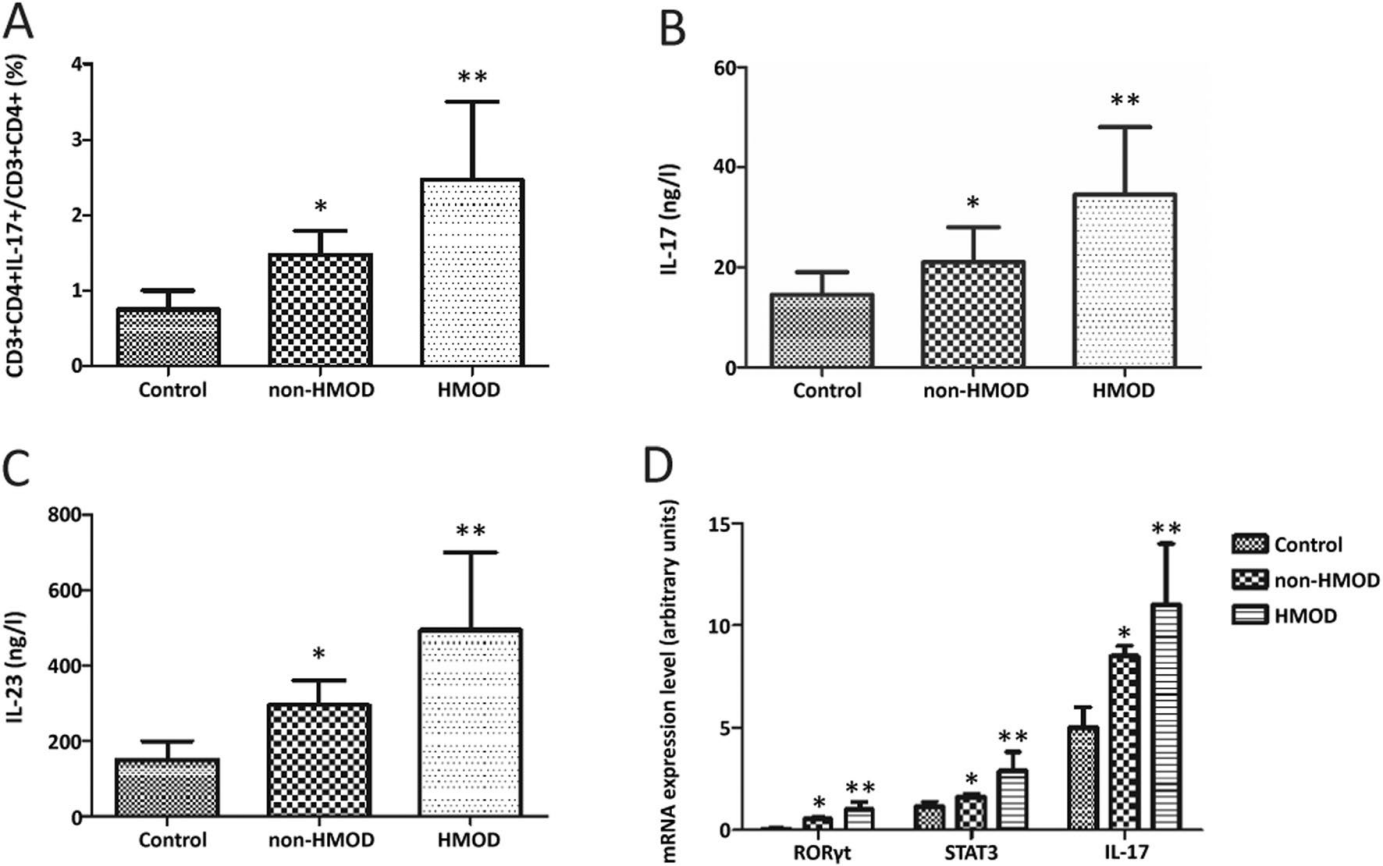
Th17 cell proportion, related cytokines and mRNA expression level in 3 groups. **A** The proportion of Th17 cells in 3 groups. The proportion of Th17 cells was significantly increased in HMOD and non-HMOD group compared with control group, while Th17 cells was also increased in HMOD group compared with non-HMOD group (*P* < 0.005). **B**, **C** The concentrations of IL-17 and IL-23 in 3 groups. IL-17 and IL-23 concentrations was significantly increased in both HMOD and non-HMOD group compared with control group, and the cytokine level in HMOD group was also higher than that in non-HMOD group (*P* < 0.005). **D** The mRNA expression level of IL-17, RORγt and STAT-3 in 3 groups. mRNA expression level of IL-17, RORγt and STAT-3 was increased in both HMOD and non-HMOD group compared with control group, and was also increased in non-HMOD group compared with control group (*P* < 0.005). Th17 cell proportion, related cytokines and mRNA expression level in control group (n = 63), non-HMOD group (n = 86) and HMOD group (n = 92) were compared by one-way analysis of variance. Bonferroni correction was used for multiple comparisons. HMOD, hypertension-mediated organ damage; RORɣt, retinoic acid-related orphan receptor γt; STAT 3, signal transducer and activator of transcription-3; IL, interleukin. *P < 0.005 when comparing with control group. **P < 0.005 when comparing with non-HMOD group

The result of flow cytometry analysis revealed that the proportion of CD3 + CD4 + IL-17A + (Th17) cells was significantly increased in HMOD and non-HMOD group compared with control group, while Th17 cells was also increased in HMOD group compared with non-HMOD group. (*P* < 0.005; Figs. 1, 2A, Table 3).

**Table 3:** Alteration of Th17 cell frequency, cytokine concentration and mRNA expression level in each group

	Control group (n = 63)	non-HMOD group (n = 86)	HMOD group (n = 92)
Th17 cell frequency (mean ± SD, %)	0.61 ± 0.46	1.35 ± 0.29*	2.94 ± 1.08**
Cytokine concentration (mean ± SD, ng/l)			
IL-17	14.05 ± 6.32	21.17 ± 9.07*	34.52 ± 19.01**
IL-23	140.10 ± 56.59	275.65 ± 283.50*	521.03 ± 169.70**
mRNA expression level (mean ± SD, arbitrary units)			
RORγt	0.05 ± 0.06	0.54 ± 0.11*	1.06 ± 0.57**
STAT3	1.15 ± 0.35	1.62 ± 0.23*	2.90 ± 1.27**
IL-17	5.06 ± 1.41	8.43 ± 0.75*	11.23 ± 4.24**

Th17 cell frequency, cytokine concentration and mRNA expression level in 3 groups were compared by one-way analysis of variance. Bonferroni correction was used for multiple comparisons.

*HMOD* hypertension-mediated organ damage, *SD* standard deviation, *RORɣt* retinoic acid-related orphan receptor γt, *STAT 3* signal transducer and activator of transcription-3, *IL* interleukin

*P < 0.005 when comparing with control group. **P < 0.005 when comparing with non-HMOD group

### Cytokine concentrations were increased in HMOD patients

To determine the relationship between Th17 cell related cytokine and HMOD, ELISA was performed to measure the concentrations of IL-17 and IL-23. The results revealed that IL-17 and IL-23 concentrations was significantly increased in both HMOD and non-HMOD group compared with control group. Moreover, the cytokine level in HMOD group was also higher than that in non-HMOD group (*P* < 0.005; Fig. 2B and C, Table 3).

### mRNA expression level of IL-17, RORγt and STAT-3

RT-PCT was used to determine the Th17 cell related mRNA expression changes in different groups. The results showed that mRNA expression level of IL-17, RORγt and STAT-3 was increased in both HMOD and non-HMOD group compared with control group, and was also increased in non-HMOD group compared with control group (*P* < 0.005; Fig. 2D, Table 3).

## Discussion

Our results showed that the proportion of Th17 cells in patients with HMOD is higher than that in non-HMOD patients and normal population. At the same time, the concentrations of IL-17 and IL-23, the Th17 cell-related cytokines, and the mRNA expression level of IL-17, RORγt, and STAT-3 in HMOD populations were significantly higher than those in the other two groups. These results were consistent with many recent studies, suggesting that Th17 cells and their related cytokines may be involved in the formation of HMOD.

T cells and their various cytokines play important roles in several hypertension models. Ang II, DOCA-salt, and excessive catecholamine stimulation can activate T cells. These cells infiltrate the perivascular regions of kidney, as well as large and small arteries [12, 13]. Monocytes/macrophages can also accumulate in these regions. Cytokines released from these cells, including IL-17, interferon-γ, TNFα, and IL-6, promote dysfunction and injury of the kidneys and blood vessels, can cause water and sodium retention and increase vascular resistance. Oxidative stress and immune activation are also linked to the formation and development of hypertension [4]. Basic researches have shown that hypertension can induce the formation of reactive oxygen species in dendritic cells and produce γ-ketoaldehydes or isoketals. These reactive oxygen species rapidly add to the protein lysine and express as neoantigens by dendritic cells, thereby activating T cells and promoting hypertension [4]. Therefore, both innate and adaptive immune system cells may be involved in the damage of hypertension target organs. Reducing the activation of these cells may reduce the damage of hypertension target organs and prevent myocardial infarction, heart failure, kidney failure and stroke.

Th17 cells, as an important type of T cells, play an important role in the formation of hypertension. IL-17 is an important cytokine of Th17 cells. Previous studies found that IL-17A secretion in mice is increased by Ang II infusion, and plasma IL-17A is also elevated in patients with hypertension in humans [3, 6]. More importantly, the blood pressure of IL-17A-deficient mice did not increase significantly after Ang II perfusion and did not show endothelial dysfunction. Consistent with this, the increased production of vascular superoxide during hypertension does not normally occur in mice lacking IL-17A. When IL-17A and TNF-α are used in human vascular smooth muscle cell culturing, IL-17A increases the expression of a variety of inflammatory cytokines and chemokines. Therefore, IL-17A may regulate the inflammatory response, leading to the accumulation of multiple immune cell subpopulations in hypertension. In addition, Amador et al. [13] found that DOCA-salt-induced hypertensive rats had increased Th17 cells and reduced regulatory T cells. Spironolactone can reverse this change and normalize the increased IL-17A mRNA expression level in heart and kidney, and anti-IL-17A antibody can also reduce blood pressure and collagen-1 level of heart and kidney. IL-17A also plays a vital role in arteriosclerosis. Ang II and DOCA-salt-induced hypertension can cause significant collagen deposition and reduced arterial compliance in the adventitia of mice [14]. However, study of Krebs et al. [2] showed protective effects of IL-17 and IL-23 in DOCA + Ang II hypertension induced renal damage. Our study verified the correlation between HMOD and Th17 cells and related cytokines in human, which was consistent with the above basic studies, but further researches are needed to reveal the causality of them.

RORγt is a key transcription factor that promotes the differentiation and proliferation of Th17 cells. IL-23 can mediate phosphorylation of STAT3 to activate STAT3, while STAT-3 can promote the expression of transcription factor RORγt, promoting differentiation and proliferation of Th17 cell [15]. Our study found that the mRNA levels of IL-17, STAT-3, and RORγt and the concentrations of IL-17 and IL-23 in the HMOD group were higher than those in the other two groups, suggesting that the increase in plasma IL-23 concentration may result in the elevation of Th17 cells in the HMOD population.

This study also has certain limitations. First, due to the small sample size, the relationship between each type of HMOD and Th17 cell ratio and related cytokines could not be analyzed separately. Second, due to the complexity of inflammation and immune regulation, the changes of Th17 cell differentiation and its related cytokines are affected by many factors. This study did not explore the impact of other clinical conditions, nor did it determine other cytokines that may affect Th17 differentiation, such as IL-6, TGF-β and so on. Third, since all patients changed their antihypertensive drugs to terazosin and/or diltiazem two weeks before admission to prepare for the tests of secondary hypertension screening, we did not record longstanding medication history, mean blood pressure level and trajectory over the years prior to enrollment, which may contribute to HMOD, immune abnormalities or inflammatory responses. Finally, this study only found the correlation between Th17 cells and related cytokines and HMOD, but did not further clarify the causal relationship and related mechanisms between them.

## Conclusions

In conclusion, the results showed that Th17 cell proportion, related cytokines and mRNA expression level in the patients with HMOD group were higher than those in the non-HMOD group and the control group. These results indicate that Th17 cells and their related cytokines may be involved in the formation of HMOD. This study provided preliminary population evidence for potential new biomarkers and immunotherapy targets in HMOD patients.

## Data Availability

The datasets used in the manuscript are available from the corresponding author upon reasonable request. All primer sequences used in RT-PCR experiments were gotten at https://www.ncbi.nlm.nih.gov/tools/primer-blast/index.cgi. Accession number: RORγt [NM_001001523.2], β-actin [NR_004845.2], STAT3 [NM_001384992.1], IL-17 [NM_002190.3].

## Abbreviations

HMOD: Hypertension-mediated organ damage
IL: Interleukin
AngII: Angiotensin II
DOCA: Deoxycorticosterone acetate
RORγt: Retinoic acid-related orphan receptor γt
STAT-3: Signal transducer and activator of transcription-3
LVMI: Left ventricular mass index
IVST: Interventricular septal thickness
LVDd: Left ventricular end diastolic diameter
PWT: Posterior wall thickness
BSA: Body surface area
LVM: Left ventricular mass
ABI: Ankle-brachial index
eGFR: Estimated glomerular filtration rate
ELISA: Enzyme-linked immunosorbent assay
RT-PCR: Real time-polymerase chain reaction
PBMCs: Peripheral blood mononuclear cells
PMA: Phorbolmyristate acetate
FITC: Fluorescein isothiocyanate
APC: Allophycocyanin
PE: Phycoerythrin
cDNA: Complementary DNA
SD: Standard deviation
ANOVA: Analysis of variance
